# Genetic education and peer support among Ashkenazi Jewish women in the United States at risk for and surviving with breast cancer

**DOI:** 10.1002/jgc4.70121

**Published:** 2025-10-12

**Authors:** Talia Zamir, Muriel R. Statman, Marcelo M. Sleiman, Duye Liu, Adina Fleischmann, Elana Silber, Kenneth P. Tercyak

**Affiliations:** ^1^ Lombardi Comprehensive Cancer Center Georgetown University Medical Center Washington DC USA; ^2^ Sharsheret Teaneck NJ USA

**Keywords:** breast/ovarian cancer, genetic education, peer support, women

## Abstract

Ashkenazi Jewish women are at significantly increased risk for hereditary breast and ovarian cancer (HBOC) due to the high prevalence of *BRCA* founder variants. Community‐based organizations (CBOs) offer culturally tailored support through programs like peer support and genetic education, but limited research has explored how these services are offered and utilized in this population. Therefore, we conducted a secondary analysis of post‐program survey data from *N* = 1054 women served by a national cancer support organization. Among high‐risk Ashkenazi Jewish women (*N* = 429), we examined patterns of genetic education and peer support program offering and utilization, patient navigation (PN) quality, care satisfaction, and health‐related quality of life (QoL). Among high‐risk Ashkenazi Jewish women, 78% were offered peer support and 33% utilized it; 59% were offered genetic education and 17% utilized it. Notably, women with poorer QoL were significantly more likely to be offered (χ^2^ = 8.06, *p* = 0.045) and utilize (*t* = −2.40, *p* = 0.009) peer support. Utilization of genetic education was more common among women with higher cancer risk (χ^2^ = 5.94, *p* = 0.049). Both programs were viewed favorably among those who participated, with users reporting increased support and decision‐making confidence. Women who were offered peer support reported significantly higher PN quality (*t* = 3.7, *p* < 0.001) and greater satisfaction with CBO care (*t* = 3.09, *p* = 0.001) than those not offered the service. Similarly, women offered genetic education reported significantly higher PN quality (*t* = 3.99, *p* < 0.001) and CBO care satisfaction (*t* = 5.38, *p* < 0.001) compared to those not offered the service. However, dual utilization of both programs was uncommon: among women offered both (*N* = 217) services, only 27% used both, suggesting potential barriers to concurrent engagement. CBO‐led peer support and genetic education may improve care satisfaction and psychosocial outcomes for Ashkenazi Jewish women navigating HBOC. Future efforts should explore integrated models that reduce barriers to dual participation and enhance continuity of care across services.

**What is known about this topic**

Ashkenazi Jewish women with pathogenic variants in *BRCA* genes face significantly increased risks for hereditary breast/ovarian cancer and experience heightened psychological burden and reduced quality of life due to complex medical decision‐making. Community‐based organizations (CBOs) have increasingly implemented culturally tailored genetic education and peer support programs, which can empower high‐risk individuals.

**What this paper adds to the topic**

This study is one of the first to examine how peer support and genetic education programs are offered to and utilized by high‐risk Ashkenazi Jewish women through a national CBO. It highlights how CBO‐led peer support and genetic education may improve healthcare satisfaction and psychosocial outcomes for special populations navigating hereditary breast/ovarian cancer, while emphasizing opportunities for concurrent program engagement.

## 1 INTRODUCTION

In the United States, the average woman has a 13% chance of developing breast cancer (BC) and a 1.1% chance of developing ovarian cancer (OC) in her lifetime (Siegel et al., 2024). However, for women with a pathogenic variant in the *BRCA* genes, this lifetime BC risk exceeds 60% (Daly et al., 2021). High‐risk women (defined as those whose individual lifetime risk is 20% or higher), such as those who carry a *BRCA* pathogenic variant, have expressed feeling overwhelmed with the options available to them, and some have difficulty understanding how *BRCA*‐associated hereditary breast/ovarian cancer (HBOC) may impact their relatives (Dason et al., 2022; Puski et al., 2018). The psychological burden of navigating these choices can impact overall well‐being and quality of life (QoL) (Dibble et al., 2022; Graves et al., 2012; Haddad et al., 2021; Isselhard et al., 2023).

While all cancer survivors may experience psychological distress, women with pathogenic variants in *BRCA* genes may experience such distress even in the absence of a cancer diagnosis (Kathleen Cunningham Foundation Consortium for Research into Familial Breast Cancer et al., 2020; Van Egdom et al., 2020). Thus, women with pathogenic variants in *BRCA* genes often express an increased need for psychoeducational support beyond what is provided in formal healthcare settings (Bertonazzi et al., 2022; Breidenbach et al., 2022). Opportunities for further education and social support have the potential to improve well‐being and medical decision‐making (Boghosian et al., 2021; Fong et al., 2017; Micińska et al., 2023).

Genetic education, delivered through genetic counseling, family history assessment, and cancer risk evaluation, offers women and their relatives the opportunity to openly discuss their medical management and receive psychosocial support (Hampel et al., 2015; Jandoubi et al., 2024). Genetics professionals can provide education about HBOC and help ensure that individuals understand guidelines for managing their and their family's risk (Berliner et al., 2021). Several cohort studies suggest that telephone education about risk between trained peers or group‐based education courses has the potential to reduce anxiety and depression (Boghosian et al., 2021; Listøl et al., 2017). Peer support programs are a prominent strategy for addressing the informational and support needs of high‐risk women by connecting them with lay health advisors who share similar life experiences and are trained in nondirective guidance (Rehberg et al., 2021). By fostering psychological (e.g., expressing feelings) and educational empowerment (e.g., answering questions and giving information), peer support can be impactful (Toija et al., 2019; Ziegler et al., 2022). Models such as peer‐to‐peer coaching or support groups have shown promising results; many participants demonstrate increased coping strategies, hope, and emotional well‐being following participation (Jansen et al., 2023; Skirbekk et al., 2018). Peer support can enhance services like genetic counseling by promoting informed decision‐making and offering emotional support beyond clinical care (Brodar et al., 2022).

Cancer‐focused community‐based organizations (CBOs) are traditionally not‐for‐profit or grassroots organizations that aim to support individuals and families affected by cancer (Feuerstein & Nekhlyudov, 2018). CBOs may offer culturally competent patient navigation (PN) to medical and non‐medical resources. Research suggests that the culturally informed care provided by CBOs can facilitate social connections, promote preventative health behaviors, and reduce stress (Levit et al., 2020; Meluch, 2022; Zisa et al., 2025). Many CBOs have attempted to amplify the power of both genetic education and peer support by establishing core programs in these areas (Brodar et al., 2022). Integrating these programs in CBOs can help encourage screening and healthy behaviors among particularly vulnerable populations that may experience disparities in access to formal healthcare services (Fernandez et al., 2014; Reyna et al., 2022). For both genetic education and peer support, previvors and survivors may be more likely to utilize programs when they can interact with lay health advisors and healthcare professionals who have similar backgrounds and culturally align with their values (Almeida et al., 2021; Bowen et al., 2023; Rehberg et al., 2021). Thus, the implementation of genetic education and peer support in communities where women may be at particularly high risk is critical for reducing barriers to both educational and social support while improving health outcomes (Guan et al., 2021; O'Neill et al., 2021; Reyna et al., 2022; Sleiman et al., 2024).

Despite the power of CBO‐based care, there is a significant gap in the literature regarding how these CBO programs are offered to and utilized by and among Ashkenazi Jewish women at risk for and surviving with BC/OC. Ashkenazi Jewish women are not only at unique biological risk, but they also may face psychosocial challenges that prevent them from seeking guidance about HBOC risk. In particular, Jewish women who follow orthodox religious customs that utilize matchmaking for potential marriages may find it difficult to seek genetic testing or information about their risk for BC/OC out of fear of stigma that could affect marriageability for them and their families (Tang et al., 2017; Trivedi et al., 2018). These women, in particular, may opt to engage with confidential CBO services where they can receive culturally informed care and speak to other Jewish women who have had similar experiences, and to do so outside of the formal healthcare system.

Toward this end, we conducted a secondary data analysis of a national not‐for‐profit CBO's one‐on‐one peer support and genetic education programming tailored for Jewish women at risk for or surviving with HBOC. In a 10‐year retrospective analysis, this study specifically evaluated high‐risk Ashkenazi Jewish women's access, utilization, QoL outcomes, and experience with these programs. Understanding their patterns is essential for informing future program development, optimizing resource allocation, and ensuring that CBOs effectively meet the needs of their communities. We sought to investigate the relationships between program participation and QoL indicators, contributing to a broader understanding of how peer support and genetic education influence health and psychosocial well‐being among cancer previvors and survivors in community settings. It was expected that the majority of eligible women would be offered these programs, although not all would opt to engage with them. Greater cancer risk and poorer QoL were expected to attenuate these observations, and women's experiences with the programs would likely be favorable.

## 2 MATERIALS AND METHODS

### 2.1 Design

This study involves a secondary analysis of self‐report survey data collected between 2014 and 2024 by Sharsheret. Sharsheret is a Jewish not‐for‐profit organization that provides personalized support, lifesaving education, financial assistance, and genetic counseling to empower those faced with or at increased genetic risk of breast cancer and ovarian cancer. While their expertise is in young women and Jewish families as related to breast cancer and ovarian cancer, Sharsheret programs serve all women and men at no cost to the community. The data were collected 30 days following participation in one or more of the organization's key programs, with the goal of ascertaining individual‐level feedback about its psychoeducational oncology programming. Participation was defined as having at least one completed encounter between Ashkenazi Jewish women and the CBO in programs focused on both peer support and genetic education. Surveys were distributed by email to all community members who engaged with the organization, including women at risk for, diagnosed with, and surviving with BC and OC. Data were collected using an online survey platform; those with incomplete surveys were prompted weekly for 3 weeks after the initial invitation and then received a final completion reminder from the organization. After that time, and if no response was received, no further survey attempts were made. This study was reviewed and approved by the host university's Institutional Review Board.

### 2.2 Measures

#### 2.2.1 Sociodemographic and clinical characteristics

Women provided self‐reported medical histories, including information on cancer previvorship and survivorship (e.g., familial and/or genetic risks for *BRCA* pathogenic variants) status related to BC, OC, or other cancers. Cancer risk and survivorship were determined by women reporting that they (1) do not carry a *BRCA* pathogenic variant or a cancer diagnosis, (2) carry a *BRCA* pathogenic variant but are not diagnosed with cancer, (3) do not carry a *BRCA* pathogenic variant but are diagnosed with cancer, and (4) carry a *BRCA* pathogenic variant and have a cancer diagnosis. Demographic details, such as age, Ashkenazi Jewish descent, marital status, race, number of children, and ages of children, were also collected (Rehberg et al., 2021).

#### 2.2.2 Patient navigation quality

PN is a process that facilitates access to quality care by facilitating one‐on‐one guidance to help patients overcome barriers to timely cancer diagnosis, treatment, and follow‐up care. PN quality was measured using a study‐specific seven‐item scale consisting of five‐point Likert ratings (1 = strongly disagree; 5 = strongly agree) to assess if the PN services received were (1) helpful, (2) informative, (3) timely, (4) effective, (5) supportive, (6) reliable, and (7) recommendable to others. These items were summed together to form a continuous PN quality score and then averaged, with higher scores indicating a higher quality PN experience (Sleiman et al., 2024; Zamir et al., 2024). The internal consistency of the PN quality measure was high (Cronbach's α = 0.93).

#### 2.2.3 Community‐based organization care satisfaction

Satisfaction with the CBO was determined through a summary score from a three‐item scale consisting of five‐point Likert ratings (1 = very dissatisfied; 5 = very satisfied). These scales assessed whether women felt satisfied with the (1) help they received and if the programs and services offered by the CBO were (2) relevant to their needs. Women also indicated their (3) overall satisfaction with the CBO. Together, these items assessed the CBO's ability to understand the needs of and provide valuable support and services to women facing cancer. This continuous measure demonstrated high reliability (Cronbach's α = 0.96) in assessing the extent to which women's needs were met and felt supported by the CBO (Van Den Berg et al., 2013).

#### 2.2.4 Health‐related quality of life

Following guidance by the Centers for Disease Control and Prevention for assessing health‐related QoL (Moriarty et al., 2003), women reported on their overall health on a continuous Likert scale item (1 = poor, 5 = excellent), as well as the total number of physically and mentally unhealthy days in the 30 days preceding the survey, and the number of days during which poor physical/mental health adversely affected their usual activities (e.g., self‐care, work, recreation).

#### 2.2.5 Program offer, utilization, and experience

Program offerings, utilization, and experience were determined by women responding to Yes/No items about whether they were offered and/or utilized two core programs: (1) a peer support network and (2) genetic education, as described below (Tercyak et al., 2015). Those who utilized a particular service were queried about their experiences with it.

The peer support network connects women who have been newly diagnosed with cancer, or are at high risk for developing BC or OC, with one‐on‐one trained volunteer peer supporters who share similar diagnoses and experiences. Peer supporters engage women over the telephone or through email and offer confidential tips for coping, perspectives on healthcare providers and treatment, and social bonds based on a shared health event. Peer support network experience was determined through a summary score from a 10‐item scale consisting of five‐point Likert ratings (1 = strongly disagree; 5 = strongly agree). These scales assessed whether women felt that peer support offered: (1) unique and (2) practical support and was (3) available, (4) a good fit, (5) appropriate, and (6) relatable, and if the supporter made them feel (7) understood, (8) supported, (9) reassured, and (10) hopeful. When summed together to form a total score, the internal consistency of the continuous peer support network satisfaction measure was high (Cronbach's α = 0.95).

The genetic education program addresses the concerns of women at higher risk of developing HBOC. This program provides women with cancer or at risk for cancer with genetic education and information related to deleterious variants in cancer predisposition genes. Women can engage with this program in multiple ways, including: (1) speaking to a certified genetic counselor about family history and cancer risk, (2) receiving a no‐cost copy of a genetic educational booklet that provides information about HBOC risk in the Ashkenazi Jewish community, and (3) connecting with a peer supporter who has first‐hand experience with similar concerns. Genetic education satisfaction was determined through a summary score from a seven‐item scale consisting of five‐point Likert ratings (1 = strongly disagree; 5 = strongly agree). These scales assessed whether women felt that the support offered by the genetic education team was: (1) informative, (2) relevant, (3) helpful, (4) timely, (5) genuine, (6) valuable, and (7) effective. When summed together to form a summary score, the internal consistency of the continuous genetic education satisfaction measure was high (Cronbach's α = 0.95).

### 2.3 Data analysis

Secondary data analyses were performed with SPSS version 29.0 and conducted in steps to examine potential differences and associations among high‐risk Ashkenazi Jewish women. High‐risk status was determined by women indicating that they were diagnosed with BC or OC and/or were carriers of a *BRCA* pathogenic variant. Descriptive statistics were generated to assess and describe all data elements and scores, and these elements and scores were checked for their categorical frequency for non‐parametric tests, and/or their range and distribution and if they met normality and other assumptions for parametric tests. Women were then grouped based on whether or not they were offered the two core programs of interest and further classified by their program utilization. Lastly, bivariate tests such as Pearson's *χ* ^2^ and *t*‐tests were used to assess the relationship between demographic and clinical characteristics with program access and utilization.

## 3 RESULTS

### 3.1 Sample characteristics

Among all women who contacted the CBO and completed a post‐program evaluation during the years of inquiry (*N* = 1054), more than one‐third self‐identified as Ashkenazi Jewish and at risk for or surviving with BC and/or OC (*N* = 429, 41%). As shown in Table 1, most high‐risk Ashkenazi Jewish women were 46 years old or older, White, and in partnered relationships; 59% carried a deleterious *BRCA* pathogenic variant and 84% were BC and/or OC survivors.

**TABLE 1:** Sample demographic and clinical characteristics (*N* = 429).

	*N*	%
Age		
18‐45 years	131	30.6
46+ years	297	69.4
Race		
White	408	95.1
Non‐White	17	3.9
Marital Status		
Partnered	308	71.8
Not partnered	107	24.9
*BRCA* and survivorship statuses		
Yes predisposition/No cancer diagnosis	69	16.1
No predisposition/Yes cancer diagnosis	176	41.0
Yes predisposition/Yes cancer diagnosis	184	42.9
Health‐related quality of life		
General health		
Excellent	67	15.6
Very good	132	30.8
Good	171	39.9
Fair/poor	57	13.3
Frequent mental distress		
Yes	78	18.2
No	351	81.8

### 3.2 Patient navigation quality

High‐risk women rated their PN quality highly, with an average score of 27.7 out of 30 (SD = 3.7), reflecting a generally positive perception of the CBO's PN services. At the individual item level, most women strongly agreed that their interactions with the CBO were both informative (76.0%) and supportive (69.7%). Additionally, a significant proportion indicated they would seek assistance from the CBO again (71.1%) or recommend its services to friends facing similar challenges (79.4%). Many women affirmed that the PN experience connected them to resources in a timely (77.9%) manner, and most women (58.3%) reported that PN helped them manage their concerns more effectively.

### 3.3 Community‐based organization care satisfaction

CBO care satisfaction among high‐risk women was also high, with an average of 13.6 out of 15 (SD = 2.1), suggesting that the CBO effectively supported and provided resources for Ashkenazi Jewish women facing BC and OC. At the item level, many women said they were very satisfied with the help that the CBO provided (63.4%) and that the rendered services met their needs (60.9%). A majority (69.1%) of women indicated that they were highly satisfied with the overall quality of care they received from the CBO.

### 3.4 Health‐related quality of life

The self‐reported item‐level assessment of health status within the sample revealed that 13.3% of high‐risk women reported being in fair or poor health (Table 1). About 18.2% of these women reported frequent mental distress (i.e., those reporting greater than 13 days of mentally unhealthy days in the past month), which is slightly higher than the national prevalence of 14.7% (Pickens et al., 2018). On average, high‐risk women reported experiencing approximately 8.0 physically unhealthy days, 6.9 mentally unhealthy days, and 5.2 activity‐limited days over the course of the past month.

### 3.5 Program engagement and experience

#### 3.5.1 Peer support engagement

**FIGURE 1:**
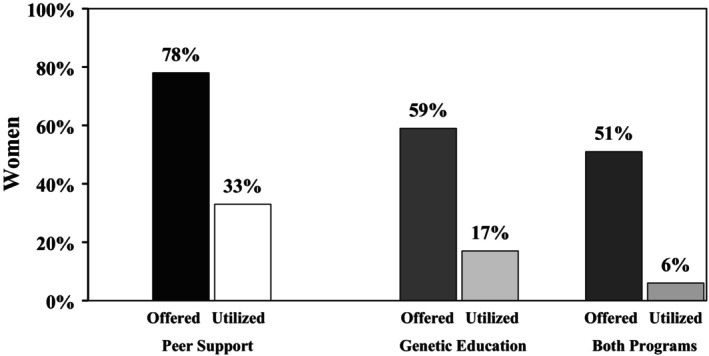
Utilization of peer support and genetic education programming.

Within the entire evaluation sample of high‐risk women (*N* = 429), the rates of offering and utilizing peer support were 78% (*N* = 334) and 33% (*N* = 142), respectively; among the subset of women offered peer support, the utilization rate was 42.5% (Figure 1). At the bivariate level, younger women (*t* = −2.64, df = 426, *p* = 0.004) and women with poorer QoL (as defined as those in less than “good” health vs. “good” health or better) (χ^2^ = 8.06, df = 3, *p* = 0.045) were more likely to be offered peer support than women without these characteristics. Similarly, those who utilized peer support (*N* = 142) were more likely to have poorer QoL than those who did not utilize it (*t* = −2.40, df = 331, *p* = 0.009).

#### 3.5.2 Peer support experience

**FIGURE 2:**
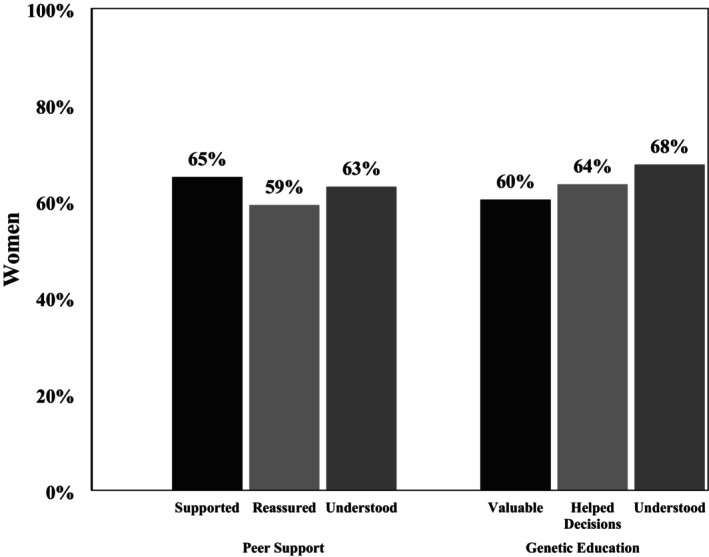
Experiences with peer support and genetic education.

Peer support ratings averaged 42.9 out of 50 (SD = 7.7) overall, indicating a favorable view of the peer support program. Among those who utilized peer support, women strongly agreed that they felt supported (65.0%), reassured (59.2%), and understood (63.1%) by their supporter (Figure 2). Women also strongly agreed that their peer supporters had similar experiences with cancer as they did (49.3%) and felt their peer supporters were a good fit for their needs (60.6%). With regard to the quality of support received, a majority of women strongly agreed that their peer supporters were available when needed (51.8%) and gave them unique (45.4%), practical (60.0%), and appropriate (56.3%) lay health advisor guidance. Most women also strongly agreed that they felt more hopeful (60.6%) about their future after speaking with their peer supporters.

#### 3.5.3 Genetic education engagement

Within the entire evaluation sample of high‐risk women (*N* = 429), 80% (*N* = 343) were aware of cancer genetic counseling as a clinical service, 69.9% (*N* = 300) had previously received a referral to cancer genetic counseling outside of the CBO, and 63.6% (*N* = 273) had engaged in counseling independently. As part of the CBO, the rates of offering and utilizing the genetic education program were 59% (*N* = 255) and 17% (*N* = 74), respectively; among the subset of women who were offered the genetics program, the utilization rate was 29% (Figure 1). There were no observed differences for which women were more or less likely to be offered the program. However, there was a trend for younger women to be more likely to be offered genetic education than older women (*t* = −1.33, df = 426, *p* = 0.09), and those with increased cancer risk were more likely to utilize it (χ^2^ = 5.94, df = 2, *p* = 0.049).

#### 3.5.4 Genetic education experience

Genetic education ratings averaged 31.4 out of 35 (SD = 4.7) overall, indicating a favorable view of the program. Women strongly agreed that their conversations with the genetic support staff were valuable (60.3%) and helped them identify their needs for making decisions about genetics (63.5%). Women strongly agreed that the support staff understood their concerns (67.6%) (Figure 2), were genuinely concerned about their well‐being (68.5%), and that the information provided was given in a timely manner (60.8%). A majority of women also strongly agreed (60.3%) that they felt their conversations about genetics were valuable, and 60.3% of women strongly agreed that they felt better after speaking with the genetics support staff.

#### 3.5.5 Dual engagement in peer support and genetic education programming

Among high‐risk Ashkenazi Jewish women who were offered peer support (*N* = 334), most (65%, *N* = 217) were also offered the genetic education program and 35% (*N* = 117) were not. By contrast, among those who utilized peer support after being offered both programs (*N* = 89), a smaller proportion (27%, *N* = 24) also utilized the genetics program, while 73% (*N* = 65) did not.

### 3.6 Associations with program engagement

In the evaluation sample of high‐risk Ashkenazi Jewish women, actual use of either the peer support program or the genetic education program was not associated with PN quality or CBO care satisfaction. However, being offered these services was linked to more positive outcomes. Women who were offered peer support reported significantly higher PN quality (*t* = 3.7, df = 417, *p* < 0.001) and greater CBO care satisfaction (*t* = 3.09, df = 412, *p* < 0.001) compared to those not offered the service. Likewise, women offered the genetic education program reported higher PN quality (*t* = 3.99, df = 412, *p* < 0.001) and higher CBO care satisfaction (*t* = 5.38, df = 417, *p* < 0.001) than those not offered the program.

## 4 DISCUSSION

This study highlights the role that CBO‐led cancer control programming plays in engaging Ashkenazi Jewish women at risk for or surviving HBOC with peer support and genetic education. About one‐third of the women who engaged with the CBO utilized peer support, which was consistent with previous estimates of peer support uptake among cancer survivors (Legg et al., 2019; Rankin et al., 2004). A majority of women who engaged in the program reported that peer support met their needs—reinforcing prior research findings that highlight the favorable impact of forming connections among women with shared lived experiences on their overall well‐being (Dunn et al., 2021; Kowitt et al., 2019; Legg et al., 2019; Sleiman et al., 2024). These findings also illustrate that women with decreased QoL were more likely to utilize peer support services, suggesting that the service is likely to encounter those who are more burdened HBOC previvors and survivors. This calls attention to the critical role peer support can play in reaching and addressing the unique psychosocial needs of individuals experiencing greater distress, positioning these programs as key intervention points for the most vulnerable members of the HBOC community. Interestingly, women offered peer support reported higher satisfaction with PN quality and CBO care than those who were not, even if they did not ultimately utilize this service. Although the direct impact of awareness of peer support services on care satisfaction has not been examined, it is possible that those who were referred to the program also received referrals to other services, which may have contributed to their positive perceptions of PN and the care they received. Ultimately, these findings underscore the potential for high‐quality PN to facilitate engagement with care among individuals with elevated HBOC risk—positioning CBO‐led programs as critical pathways for connecting high‐risk women to tailored survivorship care, including ongoing risk management, genetic counseling, and psychosocial support through peer connections.

This study also illustrated the uptake of CBO‐based genetic education within the Ashkenazi Jewish population. Less than a quarter of women who engaged with the CBO utilized the offer of genetic education programming, which is slightly lower than estimated rates of genetic counseling in clinical settings (Armstrong et al., 2015). Despite being a high‐risk population, the rates of both offering and utilizing genetic education among these women were relatively low. Those who did utilize the program were at higher risk for cancer than those who did not use the service, suggesting that the CBO may be helping to address some of the historically low rates of genetic education among higher risk women (Arun et al., 2022). Notably, most women commented that these engagements were valuable in guiding their decision‐making and improving their overall well‐being—outcomes that are also reflected in the literature (Oliveri et al., 2018; Wainstein et al., 2022; Zisa et al., 2025). Similar to peer support, women offered genetic education reported higher satisfaction with their PN and care quality from the CBO. Taken together, these findings suggest that while program uptake remains a challenge, offering services like peer support and genetic education may enhance satisfaction with care and reflect broader engagement with CBO resources.

These findings also illustrate challenges in utilizing both peer support programs and genetic education concurrently. Among those who actively utilized peer support, the majority (73%) did not utilize genetic services despite being offered both, suggesting potential barriers such as time constraints, feeling overwhelmed, perceived need, or accessibility issues (Cragun et al., 2017). It is possible that the peer support component embedded within genetic education may have been sufficient, and additional peer support was not needed. These results highlight the opportunity for CBOs to continue to develop integrated support models that seamlessly combine peer support with genetic education, ensuring that high‐risk women can access both services without added burden. Oftentimes, peer supporters are well‐equipped to communicate culturally appropriate information (e.g., about HBOC or genetic testing) to their communities, which can bridge the gap between vulnerable or underserved populations and healthcare professionals (O'Neill et al., 2021; Vadaparampil et al., 2022). While there was high satisfaction with both services separately, CBOs can look to leverage the trust and shared experience inherent in peer support relationships to enhance the effectiveness of genetic education and social connection. This approach could help ensure that more women fully benefit from both forms of support, leading to better psychosocial and health outcomes for those at risk for HBOC.

### 4.1 Limitations

This study has several limitations. First, the reliance on self‐reported data introduces the potential for subjectivity and recall bias, which may affect the accuracy of responses. However, patient‐reported outcomes remain the gold standard for understanding individuals' perspectives on their healthcare experiences and their needs for information and support. Another limitation is the potential for selection bias. The data were collected from previvors and survivors who voluntarily participated in at least one CBO‐led cancer control program and completed a post‐program survey. As a result, the experiences of those who chose not to participate or did not respond to the evaluation are not represented. Some participants may also have utilized these two core programs after completing the survey, meaning their use of peer support or genetic education could have increased post‐survey. The low utilization of genetic education services observed in this study may be influenced further by the fact that 63.6% of high‐risk women had already undergone genetic counseling before engaging with the evaluated programs, potentially skewing perceptions of the need for more assistance. Given the population attributable risk to Ashkenazi Jewish women, the goal is to engage 100% in a cancer risk assessment (King et al., 2014). Relatedly, the sample comprised women who self‐identified as Ashkenazi Jewish, thereby limiting the generalizability of the findings to the broader population affected by hereditary breast and ovarian cancer. This limitation includes, in part, the uniquely high prevalence of *BRCA* founder variants within this group, as well as relevant sociocultural factors. Future research should investigate whether these findings extend to other founder populations or to individuals with pathogenic variants outside of Ashkenazi Jewish backgrounds. Lastly, post‐program evaluations may be subject to a polarization‐response effect, where individuals with particularly strong opinions—whether positive or negative—are more likely to provide feedback, potentially influencing the overall results.

## 5 CONCLUSIONS

This study underscores the engagement and outcomes of high‐risk Ashkenazi Jewish women participating in CBO‐led peer support and genetic education programming. It provides insights to design resources in a way that supports informed decision‐making about risk management while enhancing psychological coping with HBOC prevention and treatment options. By integrating peer support with genetic education, CBOs can develop a more comprehensive, accessible, and culturally responsive care model tailored to this population's unique informational and emotional needs. PN further strengthens CBO's effectiveness by addressing both medical and psychosocial challenges. Sustained investment in these initiatives is essential—not only to improve individual well‐being but also to advance community‐level health equity by bridging gaps in access to critical services, such as cancer risk assessment and psychosocial support. By expanding and refining these efforts, CBOs can empower high‐risk Ashkenazi Jewish women with the knowledge and resources needed to take proactive control of their health, ultimately helping to reduce the broader societal impact of HBOC by improving disparities in survivorship outcomes.

## AUTHOR CONTRIBUTIONS

Substantial contribution to the conception or design of the work: Talia Zamir, Muriel R. Statman, Marcelo M. Sleiman, Jr., Duye Liu, Adina Fleischmann, Elana Silber, Kenneth P. Tercyak. Substantial contribution to the acquisition (Marcelo M. Sleiman Jr., Adina Fleischmann, Elana Silber, Kenneth P. Tercyak), analysis (Talia Zamir, Marcelo M. Sleiman Jr., Duye Liu, Kenneth P. Tercyak), and interpretation of data for the work (Talia Zamir, Marcelo M. Sleiman Jr., Duye Liu, Kenneth P. Tercyak). Drafting the work and/or revising it critically for important intellectual content: Talia Zamir, Muriel R. Statman, Marcelo M. Sleiman, Jr., Duye Liu, Adina Fleischmann, Elana Silber, Kenneth P. Tercyak. Final approval of the version to be published: Talia Zamir, Muriel R. Statman, Marcelo M. Sleiman, Jr., Duye Liu, Adina Fleischmann, Elana Silber, Kenneth P. Tercyak. Agreement to be accountable for all aspects of the work in ensuring that questions related to the accuracy or integrity of any part of the work are appropriately investigated and resolved: Adina Fleischmann, Kenneth P. Tercyak.

## CONFLICT OF INTEREST STATEMENT

Talia Zamir, Muriel R. Statman, Marcelo M. Sleiman, Jr., Duye Liu, Adina Fleischmann, Elana Silber, and Kenneth P. Tercyak declare that they have no conflicts of interest.

## ETHICS STATEMENT

Human Rights: All procedures performed in studies involving human participants were in accordance with the ethical standards of the institutional and/or national research committee and with the 1964 Helsinki Declaration and its later amendments or comparable ethical standards. IRB STUDY00008890.

Informed Consent: This is a secondary data analysis.

Welfare of Animals: This article does not contain any studies with animals.

Transparency Statements: This study was not formally registered. The analysis plan was not formally pre‐registered.

## Data Availability

De‐identified data from this study are not available in a public archive. De‐identified data from this study will not be made available due to its sensitive nature. Analytic code used to conduct the analyses presented in this study is not available in a public archive. Materials used to conduct the study are not publicly available.
